# Supplementary material for: Evaluation of Blood C Reactive Protein (CRP) and Neutrophil-to-Lymphocyte Ratio (NLR) Utility in Canine Epilepsy
Source: Vet Sci. 2024 Sep 4;11(9):408. doi: 10.3390/vetsci11090408 (PMC11436050; doi:10.3390/vetsci11090408)
Supplement: Supplementary file 1 [file vetsci-11-00408-s001.zip › vetsci-3055662-supplementary.pdf]

Table 1 – Epidemiological, clinical and diagnostic features of dogs from structural epilepsy group

| Patient | NLR value | CRP value (mg/dl) | Diagnosis | Investigations | Sex | Age (years) | Breed | Comorbidities | Type of attack | Dx |
|---|---|---|---|---|---|---|---|---|---|---|
| 1 | 4,68 | < 10 | Lafora* | Tier 1, CT, EEG | F | 7 | Beagle | No | EGTC | SE |
| 2 | 6,26 | < 10 | MUO* | Tier 1 | M | 1 | French bulldog | No | EGTC | SE |
| 3 | 9,09 | < 10 | MUO | Tier 1, CT | M | 2 | French bulldog | No | EGTC | SE |
| 4 | 5,24 | < 10 | MUO* | Tier 1 | M | 7 | French bulldog | No | EGTC | SE |
| 5 | 3,99 | < 10 | Neoplasia* | Tier 1 | F | 6 | German shepherd | No | EGTC | SE |
| 6 | 10,83 | < 10 | MUO* | Tier 1 | F | 6 | Mixed breed | No | EGTC | SE |
| 7 | 5,74 | < 10 | MUO | Tier 1, CT, EEG | F | 4 | Bichon Maltese | No | EP | SE |
| 8 | 11,68 | < 10 | MUO* | Tier 1 | M | 2 | French Bulldog | Bilateral otitis | EP | SE |
| 9 | 3,97 | < 10 | MUO* | Tier 1 | F | 2 | Yorkshire terrier | No | EP | SE |
| 10 | 17,41 | > 200 | Traumatic | Tier 1, x-ray | M | 2 | Kangal | Polytrauma | EGTC | SE |
| 11 | 3,06 | 12.4 | MUO* | Tier 1 | F | 1 | Pomeranian | No | EGTC | SE |
| 12 | 9,11 | 163.8 | MUO* | Tier 1 | F | 12 | Mixed breed | Skin lesions | EGTC | SE |
| 13 | 12,08 | 175.4 | Neoplasia* | Tier 1, EEG | M | 14 | Mixed breed | No | EP | SE |
| 14 | 6,14 | 20.4 | MUO | Tier 1, MRI, CSF, EEG | F | 3 | Pug | No | EGTC | SE |
| 15 | 13,52 | 21.1 | Vascular | Tier 1 | M | 13 | Mixed breed | No | EP | SE |
| 16 | 6,28 | 21.6 | Neoplasia | Tier 1, CT | F | 10 | Beagle | Hypothyroidism | EGTC | SE |
| 17 | 12,71 | 22.0 | MUO | Tier 1, CT | M | 6 | Bichon Maltese | No | EGTC | SE |
| 18 | 9,90 | 24.4 | Neoplasia* | Tier 1 | F | 12 | Mixed breed | No | EGTC | SE |
| 19 | 6,14 | 68.0 | MUO | Tier 1, CT | F | 3 | Pug | No | EGTC | SE |
| 20 | 6,07 | 69.7 | MUO* | Tier 1 | M | 1 | Mixed breed | No | EGTC | SE |

Legend: NLR – neutrophile – lymphocyte ratio, CRP – C reactive proteine; * - presumptive, EGTC – generalized tonicoclonic seizures; Ep – partial seizure; SE – structural epilepsy, MUO – meningoencephalitis with unknown etiology, CT – computer tomography, MRI – magnetic resonance, EEG – electroencephalography, X-Ray – radiographic examination
